# Supplementary material for: Convergent evolution of fern nectaries facilitated independent recruitment of ant-bodyguards from flowering plants
Source: Nat Commun. 2024 May 24;15:4392. doi: 10.1038/s41467-024-48646-x (PMC11126701; doi:10.1038/s41467-024-48646-x)
Supplement: Supplementary file 1 — Supplementary Information [file 41467_2024_48646_MOESM1_ESM.pdf]

Convergent evolution of fern nectaries facilitated independent recruitment of ant-bodyguards from flowering plants

Authors: Jacob S. Suissa[1]*, Fay-Wei Li[2,3], Corrie S. Moreau[4,5]

[1]Department of Ecology and Evolutionary Biology, University of Tennessee Knoxville, Knoxville, TN, USA
[2]Boyce Thompson Institute, Ithaca, NY, USA
[3]Plant Biology Section, School of Integrative Plant Science, Cornell University, Ithaca, NY, USA
[4]Department of Ecology and Evolutionary Biology Cornell University, Ithaca, NY, USA
[5]Department of Entomology, Cornell University, Ithaca, NY, USA

The following Supplementary Information is available for this article

Supplementary Notes
Supplementary Figures 1 to 14
Supplementary References

Other supporting materials for this manuscript include the following:

Supplementary Data 1 to 5
Source Data available through the GitHub repository 10.5072/zenodo.49427 (https://github.com/Suissajacob/FernNectaries).

Supplementary Notes

Datasets. We amalgamated a comprehensive dataset of nectaries in ferns, EFNs in flowering plants, plant-associated ants, and phylogenetic data of fern herbivores. We determined there are 149 nectary bearing fern species spanning 12 genera, 5 families, and 3 orders; however, our estimates are undoubtedly less than the total number of species in these categories and sampling will surely increase with additional survey. Nectaries in ferns span 1% of total species diversity, 6% of genera, 23% of families, and 43% of orders. Ferns bearing nectaries were mostly tree ferns (50.3%), then epiphytic (43.0%) and climbing (4.0%), with the fewest species being terrestrial (2.7%) (Fig. 5). Broadly, ferns are overrepresented in canopy-dwelling species (those either generating their own canopy like tree ferns, or those that live in the canopies like climbers and epiphytes). Fern lineages that have evolved nectaries include the tree ferns Cyatheaceae (*Cyathea, Alsophila, Gymnosphaera, Sphaeropteris*), the climbing ferns Schizaeaceae (*Lygodium*), the bracken fern Dennstaedtiaceae (*Pteridium*), Dryopteridaceae (*Polybotrya*), and the dominant epiphytic Polypodiaceae (*Pleopeltis*, *Campyloneuron*, *Niphidium*, *Drynaria*, *Platycerium*, *Selliguea*, and *Serpocaulon*).

We documented a total of 3913 angiosperm species with EFNs, spanning 796 genera, 119 families, and 42 orders. Interestingly, while the total angiosperm species with nectaries far outnumber ferns, we observed a similar relative proportion of species, genera, and families with nectaries. In total we found that 1% of angiosperm species have nectaries from 6% of genera, 26% of families, and 65% of orders. While not inclusive of all potential orders, the major angiosperm orders where nectaries evolved include the Malpighiales, Rosales, Fagales, Fabales, Sapindales, Brasicales, Malvales, Myrtales, Vitales, Saxifragales, Lamiales, Gentialanels, Solanales, Asterales, Dipsacales, Apiales, Ericales, Caryophyllales, Ranunculales, Arecales, Poales, Asparagales.

Using data from Nelsen *et al.,* 2018[1] we filtered their data to only include plant-associated ants (excluding leaf cutters), we were left with a total of 1341 ants from 10% of species diversity, 58% of genera (n = 173), 54% of tribes (n = 25), and 76% of subfamilies (n = 13). Nearly all major subfamilies of ants have evolved plant associations.

Fuentes-Jacques *et al.,* 2022 documented a total of 809 fern arthropod herbivores. After curation and synonymizing the taxonomy, we ended up with a total of 725 species spanning 408 genera, 74 families and 7 orders. By using this dataset as a reference, using TimeTree5[2] we acquired phylogenetic data for 581 species in the arthropod genera observed to feed on ferns. While this is clearly an underestimate and indirect, it was the first ever attempt to assemble phylogenetic data on fern arthropod herbivores. Fern herbivores include species in Hemiptera, Orthoptera, Diptera, Coleoptera, Hymenoptera, Lepidoptera, and Thysanoptera.

Nectaries in Ferns. At the species level there were an average of 85 state changes across the tree, including 17 gains and 68 losses (Supplementary Figure 1–3). The number of losses may be due to the lack of nectary observation of species within groups that have nectaries (e.g., some species in the Cyatheaceae or *Pleopeltis*). Given that most fern species do not have nectaries, the mean total time spent in each state amounts to 96.3% absent and 3.7% present. At the genus level there was an average of 13 changes across the tree with 11 gains and 2 losses (Supplementary Figure 3). The mean total time spent in each state was 97.0% nectaries absent and 3.0% present. Based on our reconstruction, ferns ancestrally lacked nectaries. Overall, our results may reflect the coding of binary nectary presence, which surely simplifies the data. While sufficient detail on the development of nectaries is currently unavailable, deeper insight may be gained from examining nectary evolution as a multistate character reflecting different developmental origins (e.g., developmental similarity to hydathodes, aerophores[3], or trichomes).

While our analyses determine that nectaries have multiple independent origins in ferns, the underlying developmental and molecular homology of these structures is uncertain. For example, nectaries in *Lygodium* (Fig. 1d) are simple gland-tipped trichomes (Supplementary Figure 4), while in Cyatheaceae and *Pteridium* (Fig. 1b, 1e) nectaries are more structured and found at pinna-rachis junctions and have been hypothesized to be homologous to aerophores [3,4]. In contrast, nectaries in *Pleopeltis* (Fig. 1c) are positionally similar to hydathodes, a homology hypothesized for EFNs in the angiosperm genus *Impatiens*

[5]. Even further, the cup-like nectary of *Drynaria speciosa* (Fig. 1a) and *Drynaria rigidula* resemble modified leaflets [6]. The phylogenetic homoplasy of nectaries in ferns is mirrored by their putative morphological and positional dissimilarity, and a detailed exploration of their molecular development may yield parallel genomic convergence. Moreover, ultimate explanations for the evolution of nectaries in ferns may hinge on ant interactions with plant wounds or as a mechanism for plants maintain hydrostatic pressure [7,8]; however, no one hypothesis is yet conclusive.

Corelated evolution between nectaries and growth habit. When analyzing growth habit data as a multistate character (terrestrial, epiphytic, climbing, and tree-habit), we found that a model assuming a hidden state and a lack of correlation between nectary presence and growth habit is supported (AICc: 827.66; Supplementary Data 1). Given our specific question regarding how ferns elevate their leaves off the forest floor, we carefully considered the appropriate coding of growth habit data. We decided to treat growth habit as a binary character, distinguishing between understory and terrestrial habitats based on whether species were in the understory (non-arboreal terrestrial species, coded as understory-dwellers) or elevate their off the forest floor (epiphytic, climbing, or arboreal, all coded as canopy-dwellers). Treating growth habit in this way is more appropriate given our specific question because we are interested in how ferns elevate their leaves off the forest floor. By categorizing growth habit as understory versus terrestrial, we emphasize the distinction between ferns that primarily grow close to the forest floor and those that have adapted to elevate their leaves off the ground. When treating growth habit as a binary character (understory vs. terrestrial) we found that a model assuming the correlated evolution of nectary presence and growth habit with a hidden rate best fit the data (AICc: 750.75; Fig. 5a–d; Supplementary Data 1).

While we have evidence to suggest correlated evolution between nectaries and growth habit, the directionality is more complicated. When nectaries are not observed transitions to nectary presence are equal and low regardless of growth habit (0.01, Fig. 5d). Moreover, when nectaries are not observed, we see much higher rates of habitat transition. In contrast, when nectaries are present, transitions between growth habitat are much lower and generally from understory to canopy (0.5). This suggests that when nectaries evolve in a particular lineage, there are less habitat transitions overall and if there are any habitat transition, they are more likely to transition to the canopy habit (Fig. 5d).

The two observed rate categories also differ substantially. Almost all transitions occur in rate category 1 compared to rate category 2 (Fig 5c,d). Rate category 2 is a much slower rate category with equal rates of transition between each state (except between nectary not observed canopy and nectary observed canopy). Based on our ancestral character state reconstruction, the majority of the fern phylogeny is in Rate category 2. Some of the major clades in Rate category 1 include the Cyatheaceae (*Cyathea*), members of the Polypodiaceae (*Drynaria*, *Pleopeltis, Campyloneurum, Serpocaulon, Platycerium*), *Elaphoglossum*, *Asplenium*, and *Lygodium*. Based on the distribution of tip states across the tree, lineages in Rate category 1 are those which either have many transitions to the epiphytic and terrestrial habitat, or have evolved nectaries (Fig. 5).

EFNs in Angiosperms. The final phylogeny used in the analyses of angiosperms included 9,589 tips with 2,636 species bearing EFNs. Given the much longer evolutionary history of ferns, we initially hypothesized that nectaries evolved in ferns before flowering plants. However, flowering plants and ferns evolved nectaries at equivalent times in the Cretaceous based on statistical analyses (Fig. 3). Across the entire flowering plants phylogeny, there was an average of 3873 state changes across the entire tree, including 1555 gains and 2318 losses. When summarizing the total time spent in each state across the tree, angiosperm evolution lacked nectaries for 75.4% of their evolutionary history. Based on our reconstruction, angiosperms ancestrally lacked nectaries (Supplementary Figure 5).

Plant-associated ants. The original dataset from Nelsen et al., 2018 [1] included a total of 1731 ant species. There were a total of 1341 ant species associated with plants and 390 unassociated with plants. Ants were ancestrally unassociated with plants, with multiple independent transitions to plant association in the Cretaceous (~100mya). Across the ant tree of life there were on average 66 stage changes to and from associations with plants. These were almost entirely driven by gains of plant associations (65), with

strikingly only a single reversion. However, given the asymmetry of gains and losses, ants only spent 68.2% of their evolutionary time with plant-associations and 31.8% without (Supplementary Figure 6).

Timing of diversification. LTT (Lineage-Through-Time) plots derived from summarizing results over the 100 stochastic character maps did not significantly differ from LTT plots derived from simple pruned phylogenies following the standard method of generating an LTT plot (Supplementary Figure 7,8). The sole discrepancy lies in the timing of emergence. In the traditional LTT diagram for ferns, the origin can be traced back to the Carboniferous period. This is attributed to the common ancestor of all lineages possessing nectaries, which can be dated approximately 375 million years ago. However, our stochastic character maps did not provide evidence for the presence of nectaries in this common ancestor.

Timing of origin denotes the oldest average potential timing of trait gain across the entire phylogenetic tree. This does not account for multiple independent origins, but rather traces the single event regardless of where it occurs across the phylogeny. Nectary origin in ferns and extrafloral nectary origin in angiosperms occurred during the same time in the middle Cretaceous (Fig. 3,4). The oldest age of nectary origin is 312.9My, very early in fern evolution; but this may be driven by the deep node of the Lygodiaceae and should be viewed cautiously. Regardless, the average age of fern nectary origin occurred remarkably late in fern evolutionary history. The median age of nectary origin in flowering plants was 132.0 Mya (Supplementary Data 3). Nectary diversification in angiosperms increased linearly and steadily since their origin with no significant lags, except a significant rate shift occurring at 7.24 Mya (Supplementary Data 4). Plant associations in ants evolved around 115.3Mya (Fig. 3, 4). We observed co-linear diversification patterns of ants and angiosperms with nectaries, especially in the Cenozoic (Fig. 4). Median time of nectary origin in ferns occurred 135.3Mya. The early origin of nectaries in ferns coincided with the rise of ants and angiosperm EFNs, but there is a lag of almost 100 million years in the diversifications of ferns with nectaries (Fig. 4). Ferns with nectaries had a diversification shift at 40 million years (Supplementary Data 3) that was 6-fold higher than previous rates. Given that most ferns with nectaries in the Cyatheaceae and Polypodiaceae, when we tease apart the data the phylogenetic patterns observed were mainly driven by these two lineages (Supplementary Figure 9). The origin of herbivore lineages which include species identified to feed on ferns dates to the Devonian (~400 Mya; Supplementary Figure 7,8). This ancient origin reflects the large phylogenetic scale of arthropod herbivores. Fern arthropod herbivores show a steady and consistent increase in diversification through time with the highest rates occurring in the Cenozoic. However, there are no noticeable lags or rate shifts (Supplementary Data 3).

Trait-based Diversification. Our analyses suggest that fern lineages with nectaries are not speciating at faster rates compared to non-nectary bearing lineages. The best fitting model of state dependent diversificaiton was a character independent model assuming four hidden states (CID-4, AICc: 34416.26), following by a CID-2 model (AICc: 34500.86), Full HiSSE model assuming two hidden states (AICc: 34501.35), BiSSE model (AICc: 36121.13; Supplementary Figure 11), and then a Null BiSSE model (AICc: 36162.57) (Supplementary Data 3).

Based on the Medusa analysis the average speciation rate of non-nectary-bearing ferns was 0.391 while the average speciation rate of nectary-bearing ferns was 0.187. Interestingly, extinction rates were much higher in non-nectary-bearing lineages (0.373) compared to nectary-bearing ones (0.086). However, many of these parameters may be driven by the observation that 99% of fern species lack nectaries. While protection from herbivory is undoubtedly beneficial for plants, the lack of a correlation between diversification and nectary presence is likely attributed to the multitude of other factors associated with the success of fern lineages (Supplementary Figure 12).

The Medusa diversification analysis revealed similar but slightly different results at the species and genus level. At the species level we found a total of 37 rate shifts including 10 downshifts and 27 upshifts ranging from 0.0049 to 3.79 (Supplementary Figure 13). Notably upshifts in diversification occurred at the base of the Eupolypods 1 and 2, 7 upshifts in the Polypodiineae, 4 upshifts in the Aspleniineae, 6 upshifts in the Pteridineae, 1 near the base of Lindsaeineae, 3 in the Cyatheales, 1 in both the Salvineales and Schizaeales, and 2 in the Hymenophyllales. At the genus level we found a total of 26 rate shifts including 7 downshifts and 19 upshifts ranging from 0.001 to 0.231 (Supplementary Figure 14). The notable

upshifts in diversification at the genus level occurred at the base of the Cyatheaceae, *Linsdaea*, *Dennstaedtia*, *Polystichum*, a subset of the Pteridaceae, Thelypteridaceae, Blechnaceae, *Selliguea* and *Pichisermollodes*, and the Grammitids. We also find an upshifts at the base of the Eupolypods 1 and 2 as well as at both common ancestors of both individual clades respectively. Of all of these upshifts, only a single rate shift event at the base of *Cyathea* seemed to be associated with the gain of nectaries. As this is a single event, it is hard to draw any broader insight.

Diversification rate shifts. To investigate the convergent evolution of ant-guarding nectaries in different taxonomic groups we employed tests of constant and lagged diversification. Model A denotes a constant diversification rate through time. Model B utilizes a Weibull distribution to explain an increasing diversification rate through time. Model C indicates a time-dependent shift in diversification at a demarked time point. To explore the correlated patterns of diversification between ferns with nectaries, fern herbivores, angiosperms with nectaries and ants forming associations with plants, we fit models A–C to each dataset. Ferns with nectaries show a marked temporal rate shift, with a 6-fold increase in diversification after 40 million years (AIC: 1131.01). While fern herbivores are not diversifying at a constant rate, they do not have a marked increased diversification at 40 million years (the time of fern nectary diversification; Supplementary Data 3). Instead, fern herbivores are diversifying at rates that increase to the present with no noticeable lags or rate shifts. We do not find that underlying diversification models correlate between the fern herbivore and fern-nectary phylogenies (Supplementary Data 3). Model B is marginally more supported compared to Model B for Angiosperms with EFNs (Supplementary Data 3) however the time point breaks most supported is 7.24 million years ago. This suggests that angiosperms with ENFs are likely diversifying following patterns of increased diversification rates through time. Likewise, ants associated with plants follow a similar pattern of increasing diversification through time (Supplementary Data 3).

Supplementary Figures

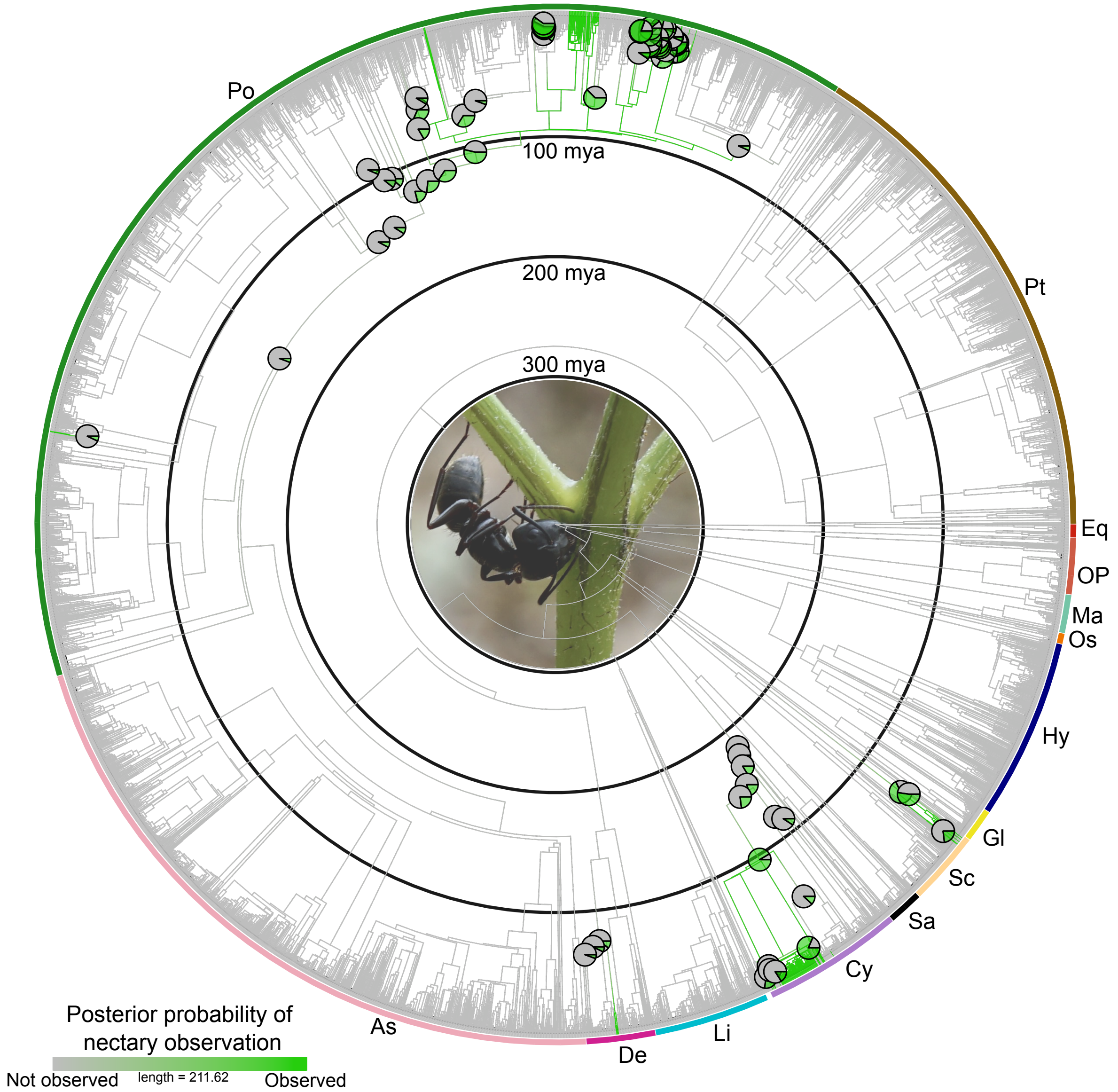

Figure. 1. The ancestral character estimation of nectary evolution across all fern species. Nectaries evolved at least seven times independently and the ancestral character estimation suggests that nectaries had the potential to evolve in the Cretaceous. However, the majority of nectary gains did not occur until the Cenozoic. Ancestral character estimation was implemented using Bayesian stochastic character mapping. Pie charts at nodes along the time-calibrated fern phylogeny represent ancestral states calculated as the marginal posterior probability of nectary presence (green) or unobserved (gray). Colors along the branches represent estimated character states summarized across 100 randomly selected posterior samples of character histories from 1000 stochastic character maps. Bars at tips indicate the species character state for nectary presence or absence. Bars along the perimeter of the reconstructed tree and bolded letters indicate major fern clades. Light pink: Aspleniineae, Green Polypodiineae, Brown: Pteridineae, Red: Equisetales, Dark orange: Ophioglossales & Psilotales, Teal: Marattiales, Orange: Osmundales, Blue: Hymenophyllales, Yellow: Gleicheniales, Cream: Schizaeales, Black: Salviniales, Purple: Cyatheales, Light blue Lindsaeineae, Pink: Dennstaedtiineae. Black inlaid circles indicate 100-million-year time intervals. Pie charts with confidence intervals higher than 95% were dropped for both observed and unobserved nodes. Photo inset of an ant feeding on fern nectar taken by JSS.

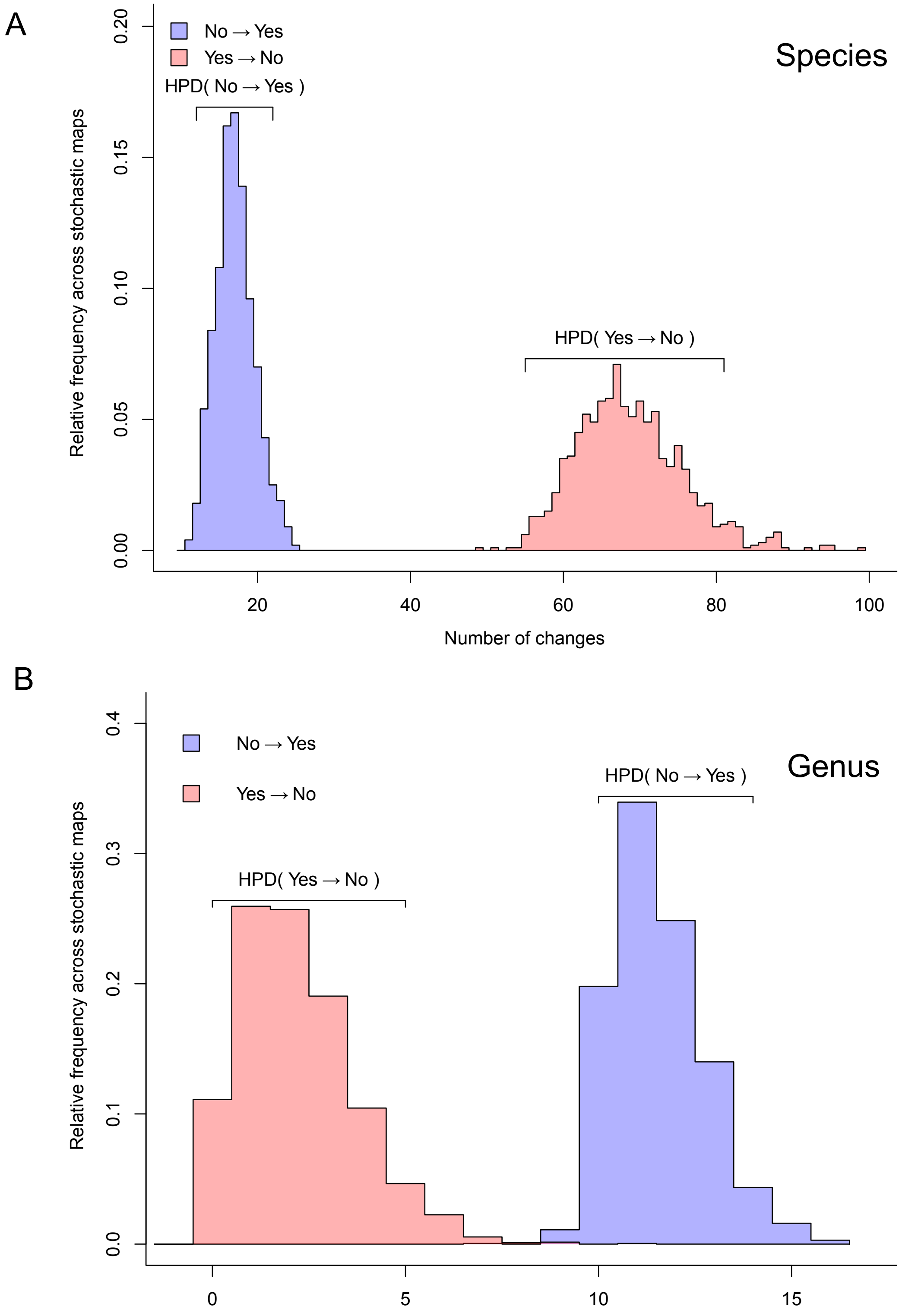

Figure. 2. Relative frequency of the number of transitions summarized across 1000 stochastic character maps exploring the evolution of fern nectaries for A. species level reconstructions, and B. genus level reconstructions. All losses are colored in red and all gains are colored in blue.

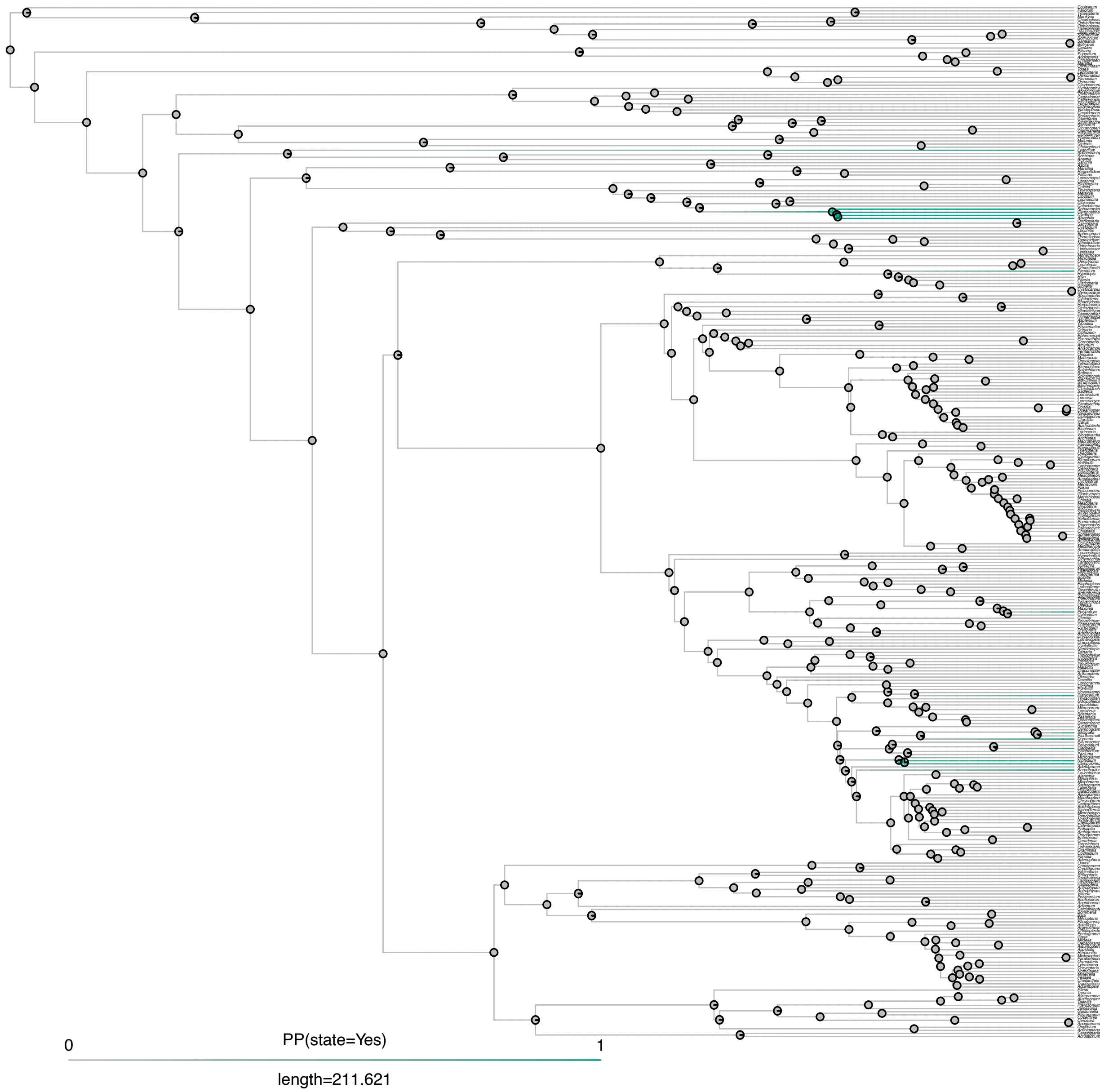

Figure. 3. The ancestral character estimation of fern nectary evolution at the genus level. Nectaries evolved at least seven times across fern genera. The majority of nectary gains did not occur until the Cenozoic. Ancestral character estimation was implemented using Bayesian stochastic character mapping. Pie charts at nodes along the time-calibrated fern phylogeny represent ancestral states calculated as the marginal posterior probability of nectary presence (green) or unobserved (gray). Colors along the branches represent estimated character states summarized across 100 randomly selected posterior samples of character histories from 1000 stochastic character maps.

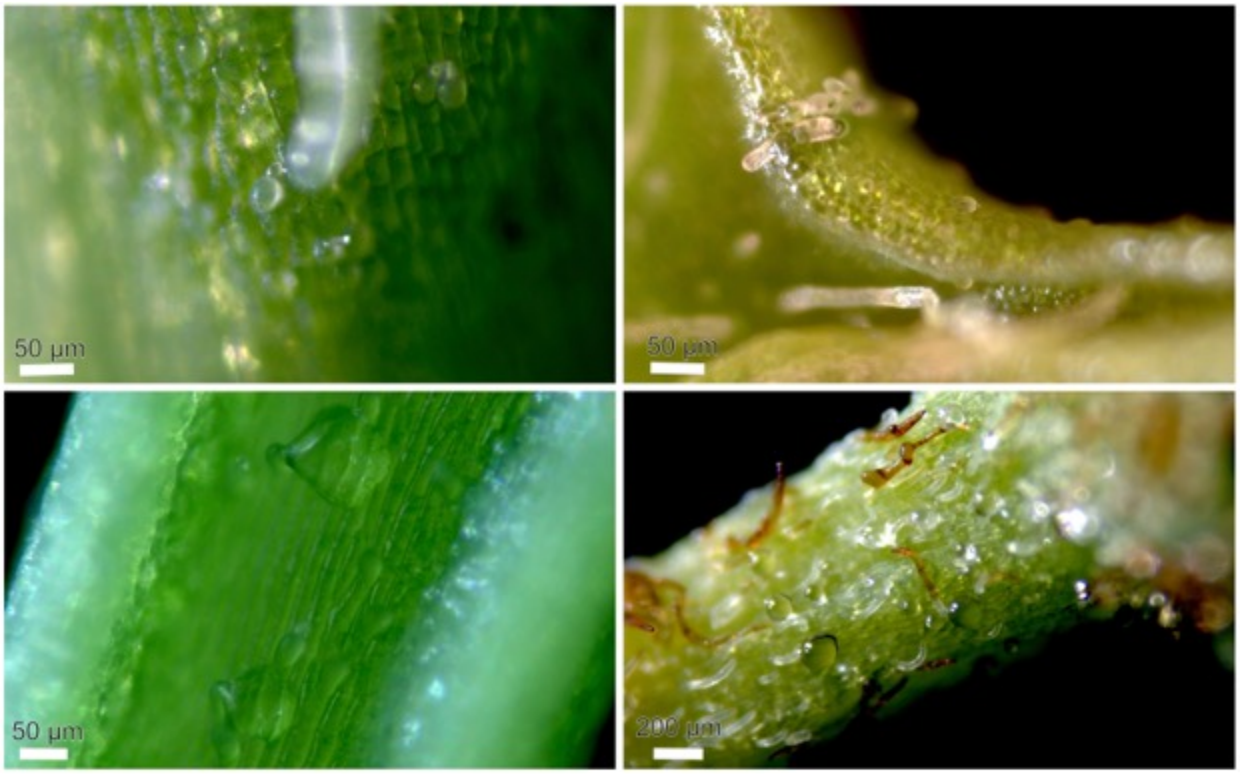

Figure 4. Four images of Lygodium pinna showing nectar secreted from gland tipped trichomes. The presence of sugar in the secreted liquid has been conducted by previous analyses [9].

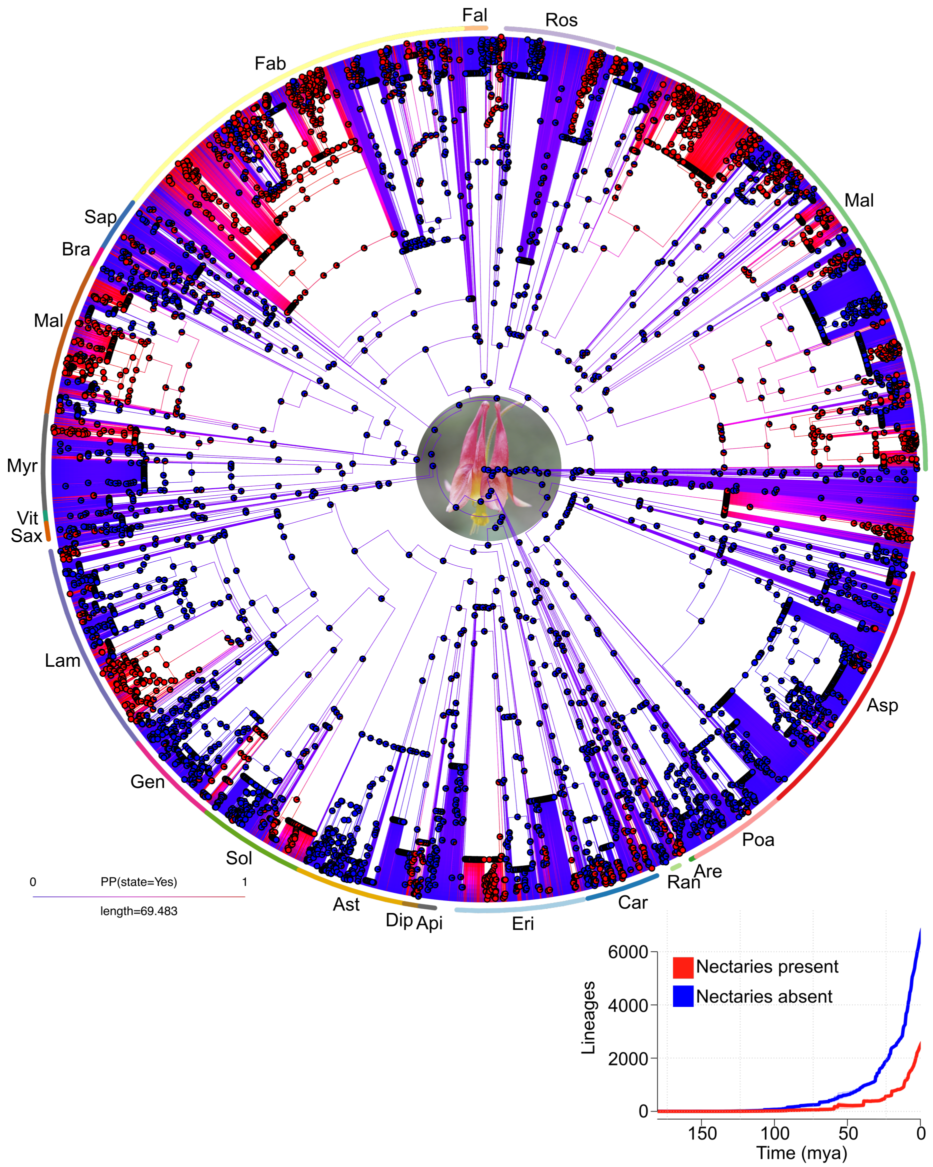

Figure. 5. The ancestral character estimation of EFNs in angiosperms. Ancestral character estimation was implemented using Bayesian stochastic character mapping. Pie charts at nodes along the time-calibrated fern phylogeny represent ancestral states calculated as the marginal posterior probability of EFN presence (red) or unobserved (blue). Colors along the branches represent estimated character states summarized across 100 of the 1000 posterior samples of character histories. Bars along the circumference of the reconstructed tree and bolded letters indicate major angiosperm clades. Mal: Malpighiales, Ros: Rosales, Fal: Fagales, Fab: Fabales, Sap: Sapindales, Bra: Brassicales, Mal: Malvales, Myr: Myrtales, Vit: Vitales, Sax: Saxifragales, Lam: Lamiales, Gen: Gentianales, Sol: Solanales, Ast: Asterales, Dip: Dipsacales, Api: Apiales, Eri: Ericales, Car: Caryophyllales, Ran: Ranunculales, Are: Arecales, Poa: Poales, Asp: Asparagales. Inset in the bottom left depicts the lineage through time plot derived from summarizing the 100 stochastic character maps. Photo inset of a flower of Aquilegia canadensis taken by JSS.

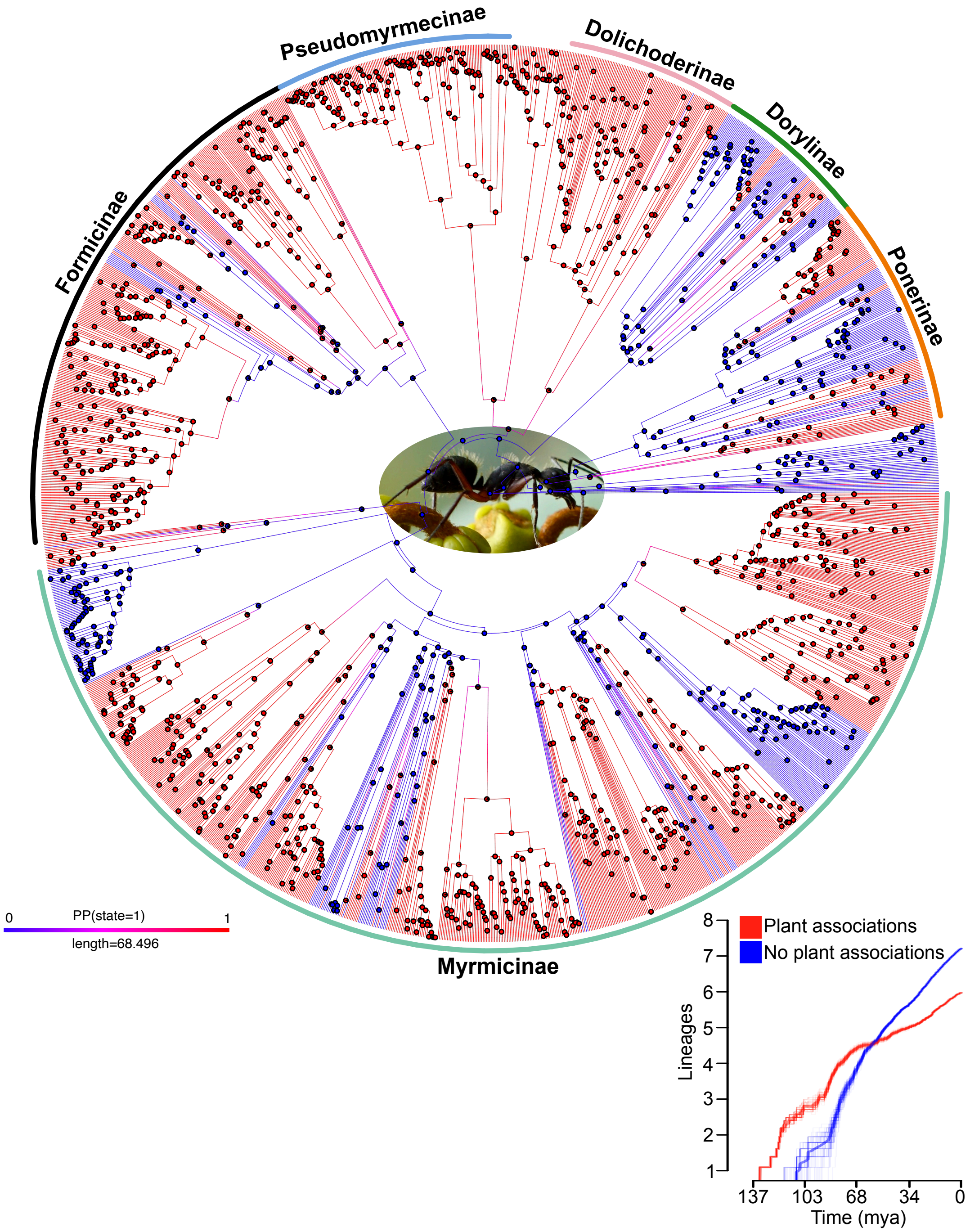

Figure. 6. Figure illustrating the ancestral character estimation of ants that associate with plants. Ancestral character estimation was implemented using Bayesian stochastic character mapping. Pie charts at nodes along the time-calibrated fern phylogeny represent ancestral states calculated as the marginal posterior probability of plant-association presence (red) or absent (blue). Colors along the branches represent estimated character states summarized across 100 of the 1000 posterior samples of character histories. Bars along the circumference of the reconstructed tree indicate major ant subfamilies. Inset in the bottom left depicts the lineage through time plot derived from summarizing the 100 stochastic character maps. Photo inset of ant taken by JSS.

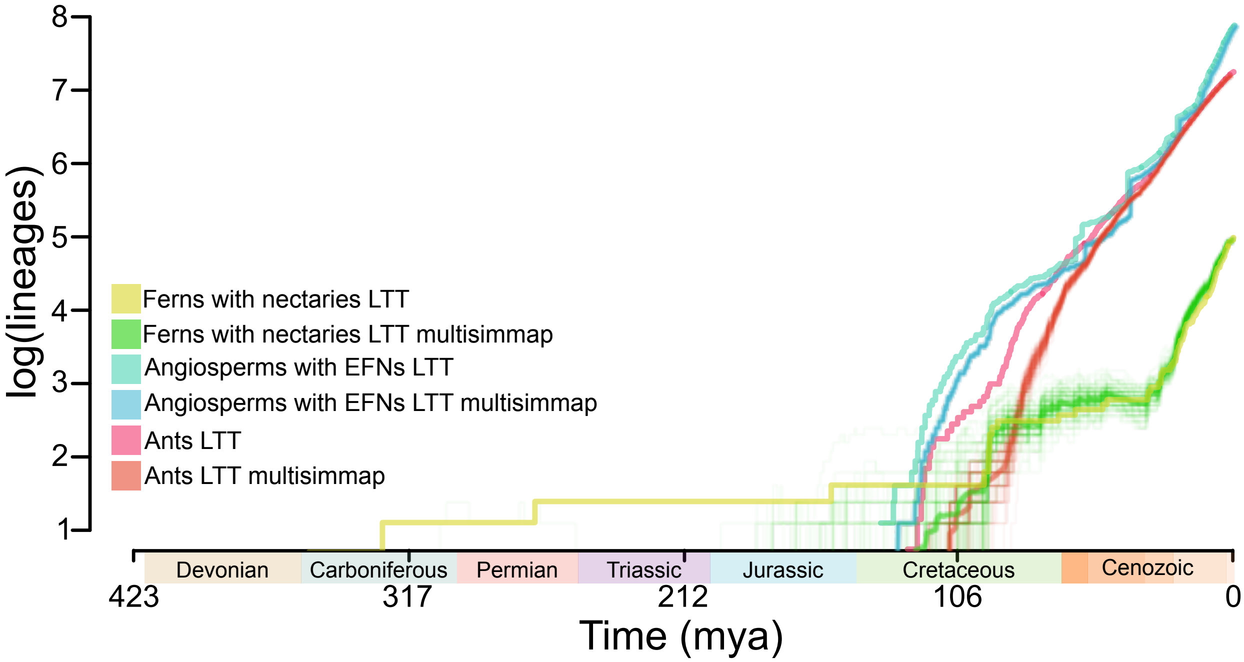

Figure. 7. Lineage-through-time (LTT) plots for ferns with nectaries, angiosperms with EFNs and Ants associated with plants. LTT plots derived based on the traditional method (simply using a phylogeny) or from 100 sampled stochastic character maps. Each thin line represents a single LTT plot for 1/100 stochastic character maps, the larger line represents the average LTT across all maps. The LTT plot shows the cumulative number of lineages through time for each group, as indicated by the colored lines on the graph. The x-axis represents time in millions of years before present. LTT plots derived from the traditional method do not significantly differ from those derived from stochastic character mapping. The only major difference is in the origin of ferns with nectaries. This carboniferous origin in the original LTT plot is due to the deep common ancestor of all lineages with nectaries but likely does not represent the actual origin of nectaries in the lineage.

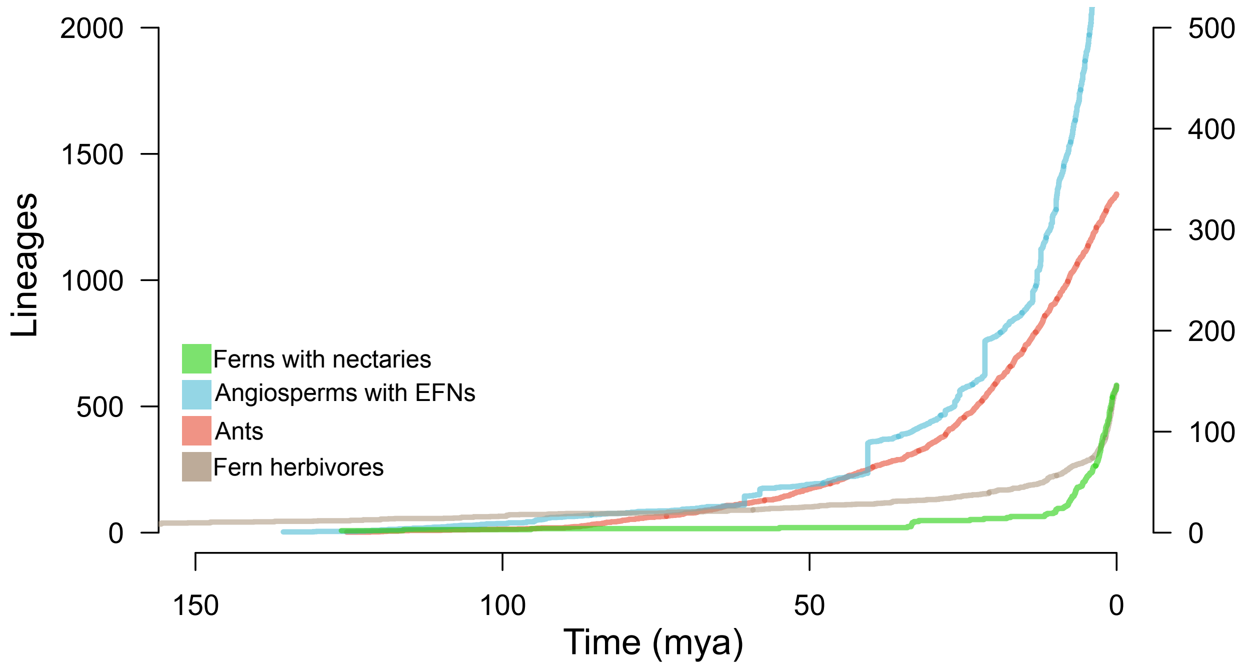

Figure. 8. Lineage-through-time (LTT) plots for ferns with nectaries, angiosperms with EFNs, and fern arthropod herbivores, and ants associated with plants. LTT plots derived based on the traditional method (simply using a phylogeny) and are not log scaled. The left y axis represents lineages for ants, herbivores, and angiosperms, while the left y axis represents fern lineages. The LTT plot shows the cumulative number of lineages through time for each group, as indicated by the colored lines on the graph. The x-axis represents time in millions of years before present.

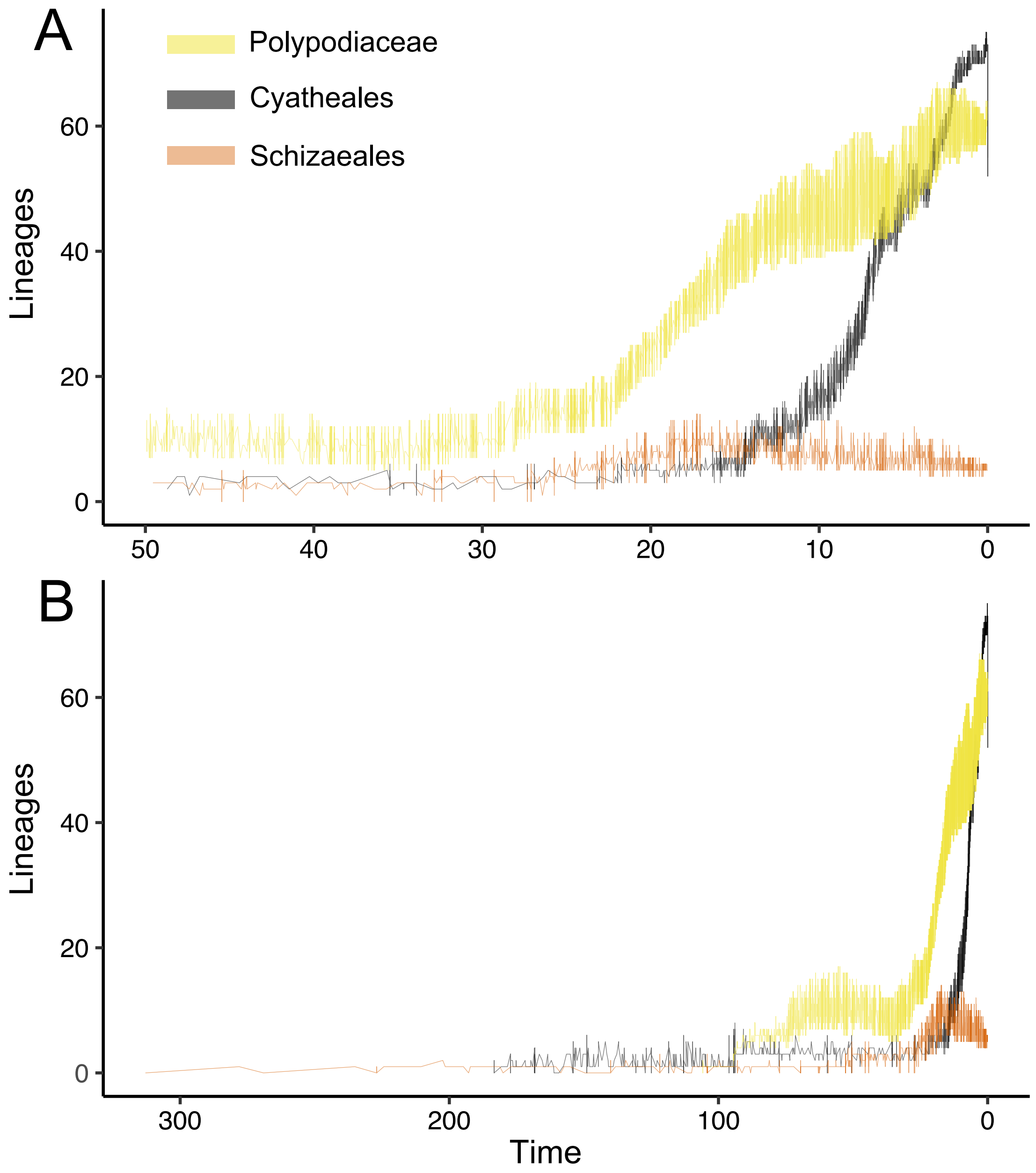

Figure 9. Lineage-through-time (LTT) plots for three major fern clades with nectaries (Polypodiaceae, Cyatheales, and Schizaeales). Plots A and B are the same graph but with different X-axes. A. LTT plot with truncated X-axis depicting time in the Cenozoic. B. LTT plot with X-axis depicting the time since potential nectary gain in the Scizaeales. LTT plots derived from 100 sampled stochastic character maps. Each thin line represents a single LTT plot for 1/100 stochastic character maps. The LTT plot shows the cumulative number of lineages through time for each group, as indicated by the colored lines on the graph. The x-axis represents time in millions of years before present. LTT plots derived from the traditional method do not significantly differ from those derived from stochastic character mapping.

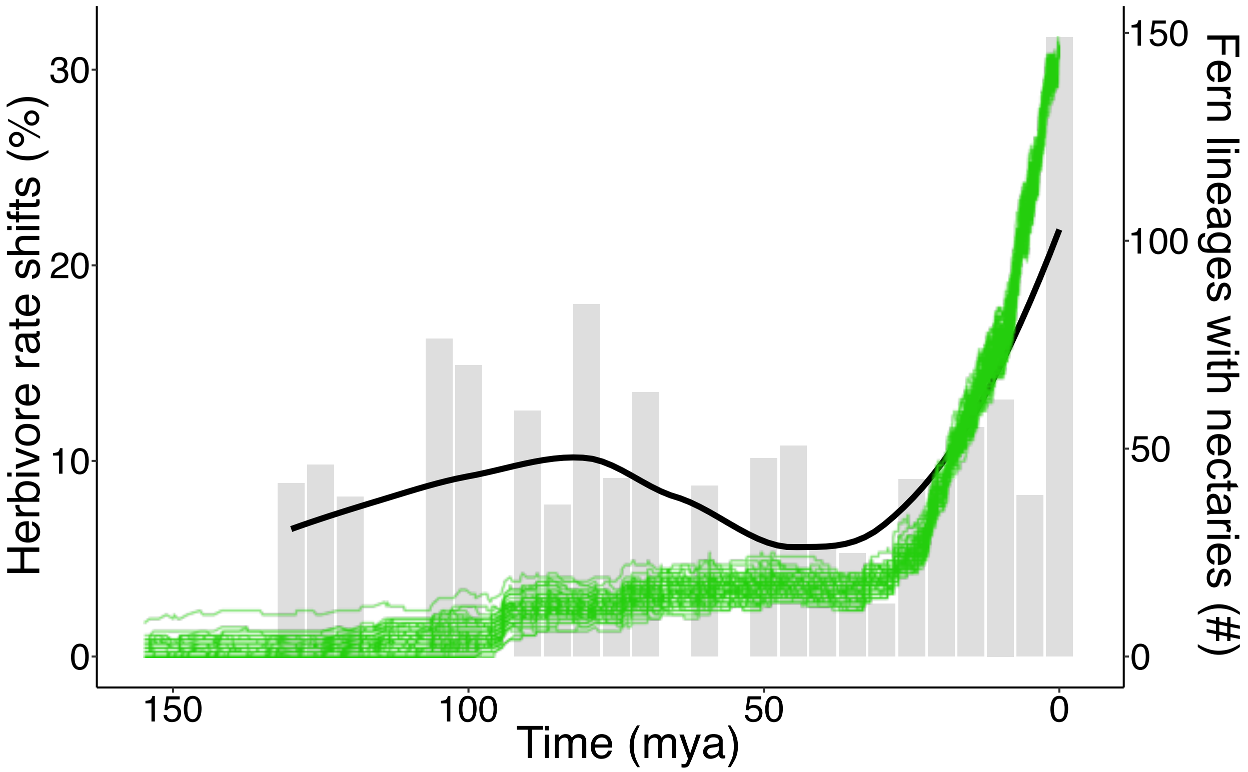

Figure 10. Summarized rate shifts (upshifts only) from the Medusa analysis across the fern-feeding herbivore phylogeny (gray bins and black curve) and the diversification patterns of ferns with nectaries (green lines). Rate shifts, shown as gray bins, represent the proportion of total shifts occurring within temporal sliding windows. The black curve depicts the best fit line of the distribution of these rate shifts. The multiple green lines represent Lineage-Through-Time (LTT) plots, each derived from an individual realization of 100 stochastic character maps of nectary presence across ferns (as in Fig. 3). The left y-axis corresponds to the percentage of total shifts occurring in each time window, illustrating the temporal distribution of rate shifts. The right y-axis denotes the number of fern lineages at each time point, providing a visualization of fern diversification patterns.

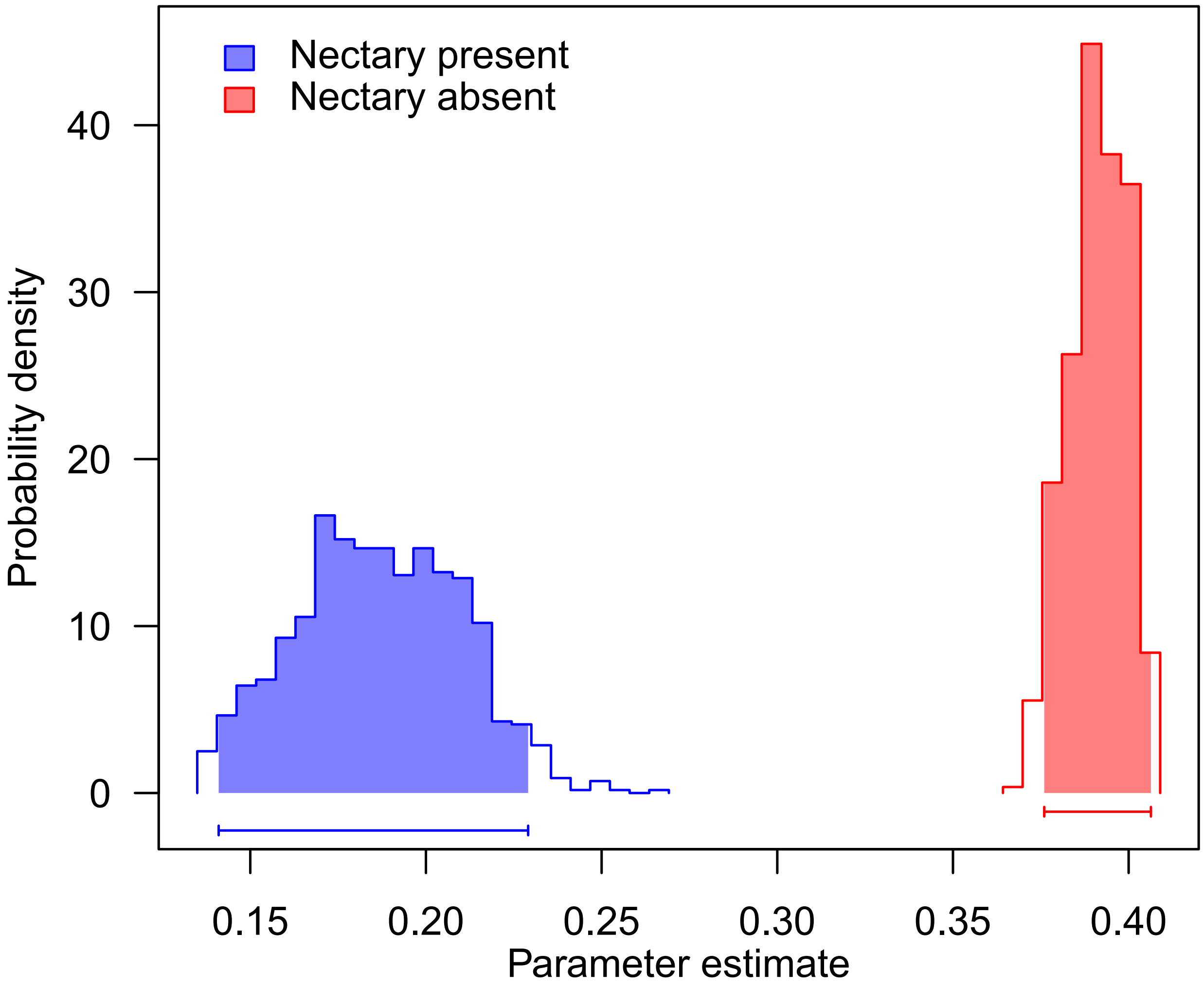

Figure. 11. Posterior distribution of speciation rates for nectary bearing species (blue) and non-nectary bearing species (red), calculated with BiSSE, as implemented in diversitree.

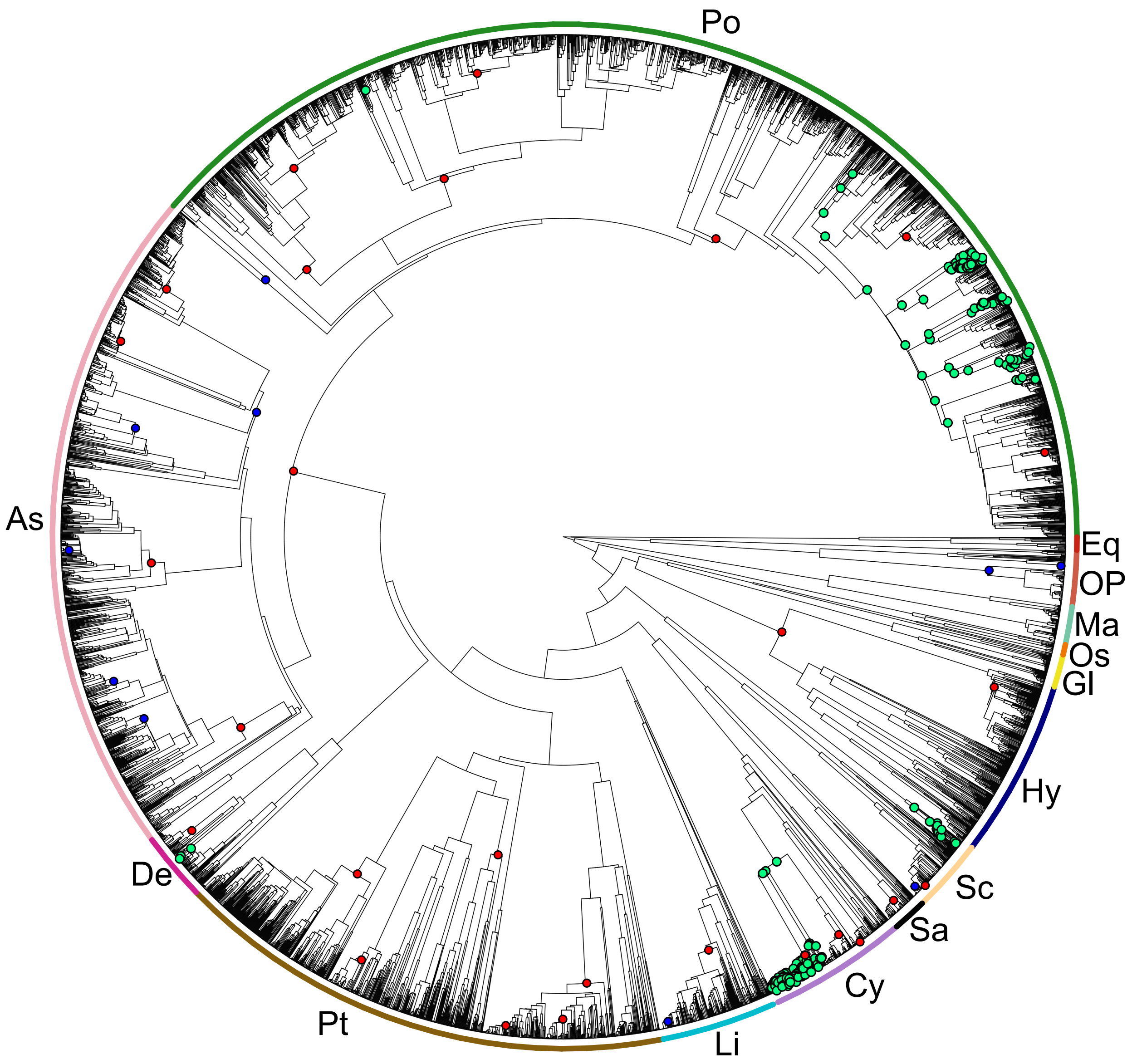

Figure. 12. Fern phylogeny showing nectary gains at nodes with at least 75% posterior probability of nectary presence (green dots), 27 diversification rate upshifts (red dots), and 10 diversification downshifts (blue dots) from the MEDUSA analysis. Only a single gain of nectaries is correlated with a rate shift at the base of *Cyathea*. Bars along the perimeter of the reconstructed tree and bolded letters indicate major fern clades. Light pink: Aspleniineae, Green Polypodiineae, Brown: Pteridineae, Red: Equisetales, Dark orange: Ophioglossales & Psilotales, Teal: Marattiales, Orange: Osmundales, Blue: Hymenophyllales, Yellow: Gleicheniales, Cream: Schizaeales, Black: Salviniales, Purple: Cyatheales, Light blue Lindsaeineae, Pink: Dennstaedtiineae.

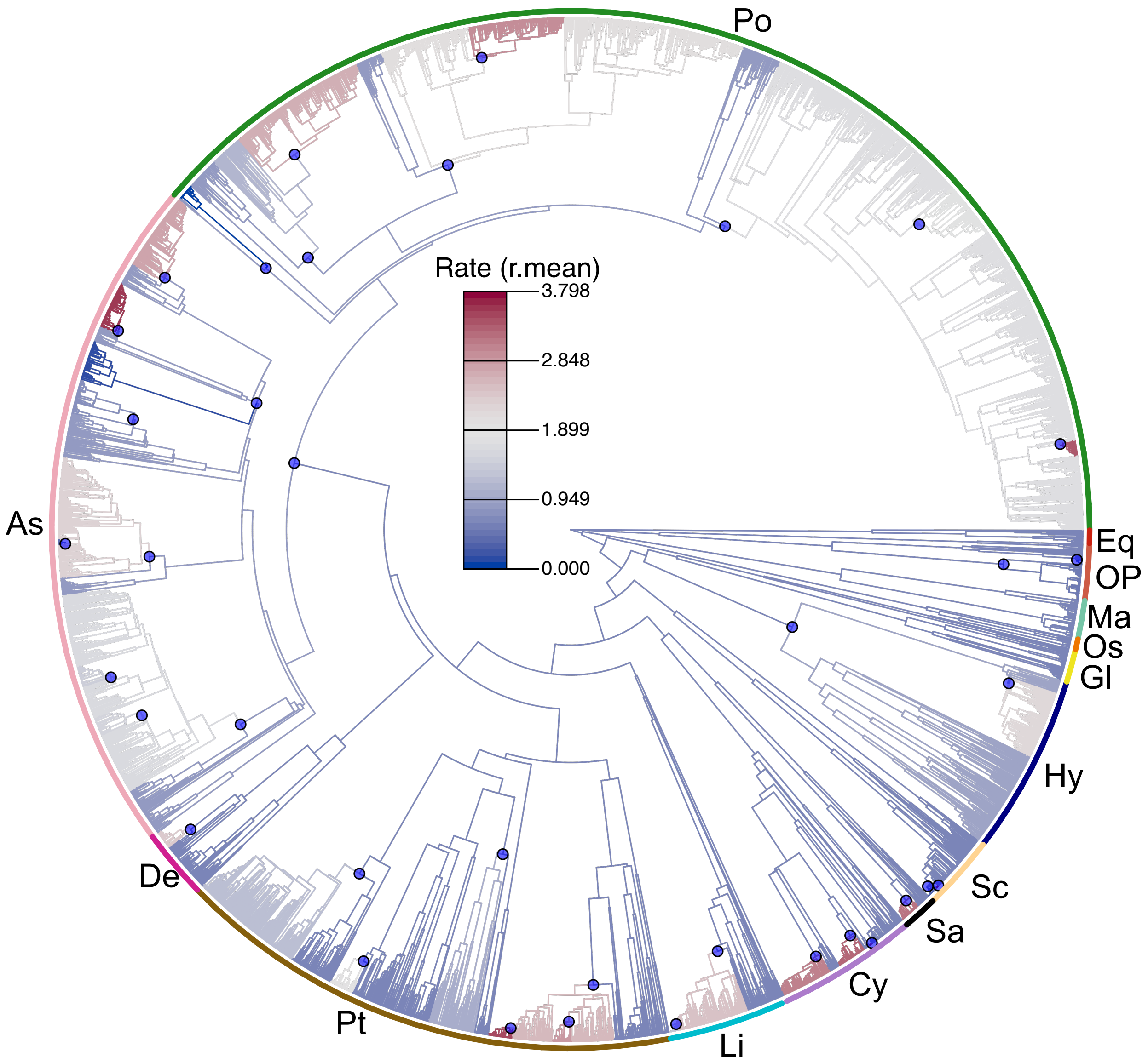

Figure. 13. Diversification rate analysis for the entire fern phylogeny implemented in Medusa. Red bars indicate lineages with higher diversification rates while blue indicates low diversification rates. Rate shifts (both upshifts and downshifts) are denoted with blue dotes. Bars along the perimeter of the reconstructed tree and bolded letters indicate major fern clades. Light pink: Aspleniineae, Green Polypodiineae, Brown: Pteridineae, Red: Equisetales, Dark orange: Ophioglossales & Psilotales, Teal: Marattiales, Orange: Osmundales, Blue: Hymenophyllales, Yellow: Gleicheniales, Cream: Schizaeales, Black: Salviniales, Purple: Cyatheales, Light blue Lindsaeineae, Pink: Dennstaedtiineae.

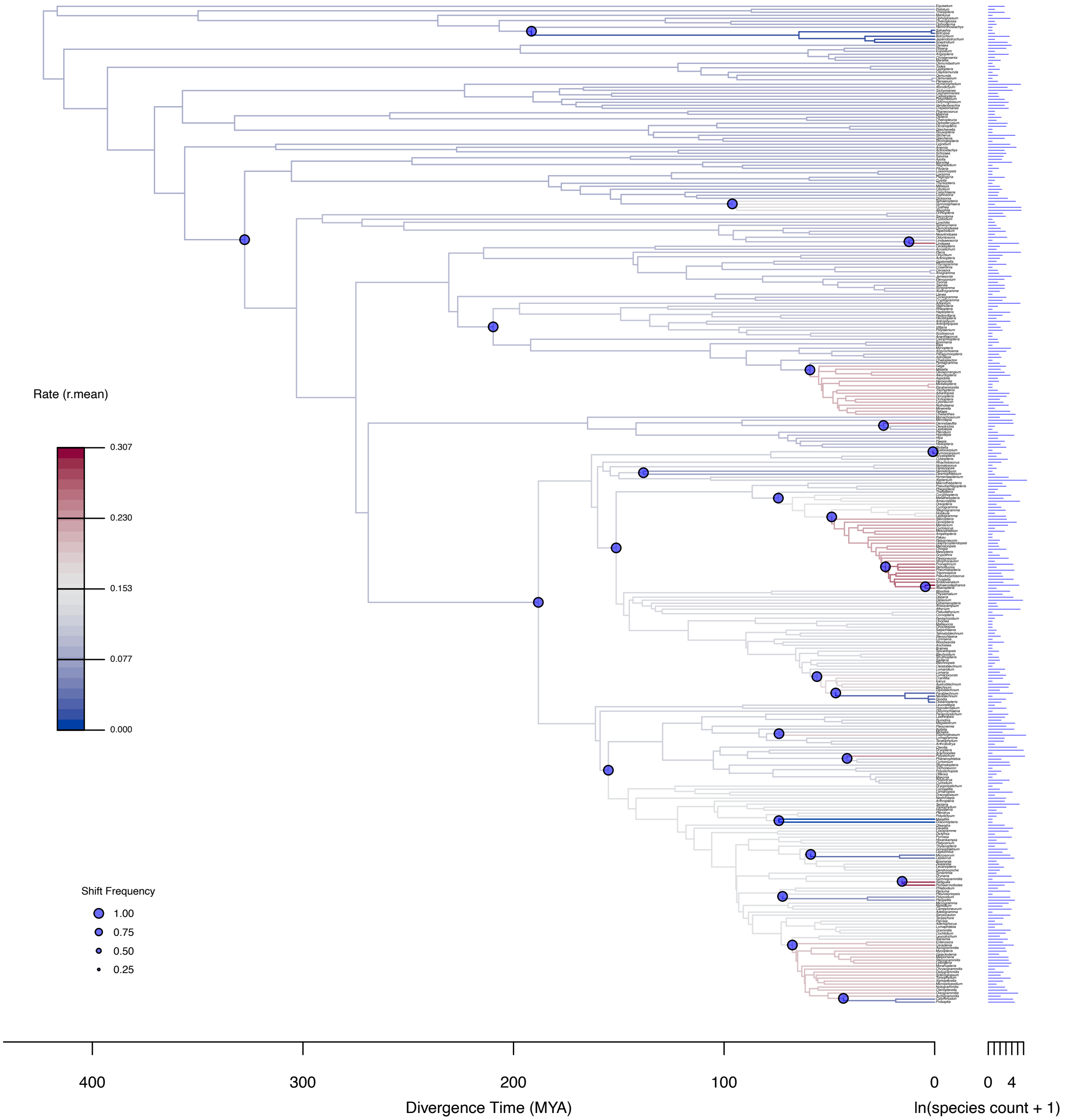

Figure. 14. Genus level diversification rate analysis implemented in Medusa. The fern phylogeny was pruned to 1 tip per genus and species richness values were input and displayed as blue bars on the right of the phylogeny (log scale). X-axis is absolute time. Red bars indicate lineages with higher diversification rates while blue indicates low diversification rates. Rate shifts (both upshifts and downshifts) are denoted with blue dotes.

Supplementary References

1. Nelsen, M. P., Ree, R. H., & Moreau, C. S. Ant–plant interactions evolved through increasing interdependence. *Proceedings of the National Academy of Science* **115**, 12253–12258 (2018).
2. Kumar, S. *et al.* TimeTree 5: An Expanded Resource for Species Divergence Times. *Mol. Biol. Evol.* **39**, (2022).
3. Moran, R. C. Remarks on aerophores and the relationship between sterome and stomata in ferns. *Brittonia* **74**, 123–147 (2022).
4. Lloyd, F. E. The extra-nuptual nectaries in the common brake, Pteridium aquilinum. *Science* **13**, 885–890 (1901).
5. Elias, T. S. & Gelband, H. Morphology, Anatomy, and Relationship of Extrafloral Nectaries and Hydathodes in Two Species of Impatiens (Balsaminaceae). *Bot. Gaz.* **138**, 206–212 (1977).
6. Potes, A. Comparative Anatomy of the Nectaries of Aglaomorpha and Drynaria (Polypodiaceae). *amfj* **100**, 80–92 (2010).
7. Zimmermann, J. Uber die extrafloralen Nektarien der Angiosperm. *Bot. Cent.bl.* **49**, 99–196 (1932).
8. De la Barrera, E. & Nobel, P. S. Nectar: properties, floral aspects, and speculations on origin. *Trends Plant Sci.* **9**, 65–69 (2004).
9. Sebesta, N., Jones, I. M. & Lake, E. C. First Report of Foliar Nectar Production by Lygodium microphyllum (Lygodiaceae), an Invasive Fern in Florida. *Am. Fern J.* (2018).
