# Supplementary material for: Convergent evolution of fern nectaries facilitated independent recruitment of ant-bodyguards from flowering plants
Source: Nat Commun. 2024 May 24;15:4392. doi: 10.1038/s41467-024-48646-x (PMC11126701; doi:10.1038/s41467-024-48646-x)
Supplement: Supplementary file 3 — Description of Additional Supplementary Files [file 41467_2024_48646_MOESM3_ESM.pdf]

Description of Additional Supplementary Files

Supplementary Data 1. Breakdown of the logliklihood and AIC for each tested model of nectary presence and growth habit ordered by AICc. Nectary presence was treated as a binary character (observed/unobserved). Growth habit was treated in two ways. First as a binary character (terrestrial/arboreal). Or as a multistate character where arboreal was broken up into epiphytic, tree habit, or climbing.

Supplementary Data 2. Mean number of transitions between states depending on the rate category. Data generated from summarizing information of the 100 stochastic character maps using the transition matrix output from CorHMM.

Supplementary Data 3. Summarized timing of major events across lineages and traits. Data generated from summarizing the time of trait gains across all simulated character histories.

Supplementary Data 4. Summary of the analysis of diversification models. Model A assumes constant diversification through time with no rate shifts or lags. Model B assumes diversification follows a Weibull function. Model C assumes that diversification is time-dependent and shifts with a specific breakpoint. Models are fitted by maximum likelihood. δ = rate of diversification, $\delta 1$ is the rate shift after the break point (younger than the time point) and $\delta 2$ is the rate shift before the time break (older than the time point). β and α are parameters in the Weibull function.

Supplementary Data 5: Dataset including Fern species with nectaries and growth habit, Angiosperm species with nectaries, Ant species identified to associate with plants, and Taxa returned from TimeTree5 within the genera identified to host fern arthropod herbivores. Each taxon may not be a direct fern herbivore but represents a close relative with molecular data used to construct the phylogeny. HiSSE model output is also a page in the supporting dataset.
