# Supplementary material for: Convergent evolution of fern nectaries facilitated independent recruitment of ant-bodyguards from flowering plants
Source: Nat Commun. 2024 May 24;15:4392. doi: 10.1038/s41467-024-48646-x (PMC11126701; doi:10.1038/s41467-024-48646-x)
Supplement: Supplementary file 6 — Reporting Summary [file 41467_2024_48646_MOESM6_ESM.pdf]

nature portfolio

Corresponding author(s): Jacob S. Suissa

Last updated by author(s): Apr 28, 2024

Reporting Summary

Nature Portfolio wishes to improve the reproducibility of the work that we publish. This form provides structure for consistency and transparency in reporting. For further information on Nature Portfolio policies, see our Editorial Policies and the Editorial Policy Checklist.

Statistics

For all statistical analyses, confirm that the following items are present in the figure legend, table legend, main text, or Methods section.

n/a	Confirmed	
☐	☒	The exact sample size (*n*) for each experimental group/condition, given as a discrete number and unit of measurement
☒	☐	A statement on whether measurements were taken from distinct samples or whether the same sample was measured repeatedly
☐	☒	The statistical test(s) used AND whether they are one- or two-sided<br>*Only common tests should be described solely by name; describe more complex techniques in the Methods section.*
☐	☒	A description of all covariates tested
☐	☒	A description of any assumptions or corrections, such as tests of normality and adjustment for multiple comparisons
☐	☒	A full description of the statistical parameters including central tendency (e.g. means) or other basic estimates (e.g. regression coefficient) AND variation (e.g. standard deviation) or associated estimates of uncertainty (e.g. confidence intervals)
☐	☒	For null hypothesis testing, the test statistic (e.g. *F*, *t*, *r*) with confidence intervals, effect sizes, degrees of freedom and *P* value noted<br>*Give P values as exact values whenever suitable.*
☒	☐	For Bayesian analysis, information on the choice of priors and Markov chain Monte Carlo settings
☒	☐	For hierarchical and complex designs, identification of the appropriate level for tests and full reporting of outcomes
☒	☐	Estimates of effect sizes (e.g. Cohen's *d*, Pearson's *r*), indicating how they were calculated

Our web collection on statistics for biologists contains articles on many of the points above.

Software and code

Policy information about availability of computer code

Data collection	Microsoft Excel and Google docs were used to sore data during collection. TimeTree was used to generate phylogenetic data on fern-arthropod herbivores. No additional software was used during data collection.
Data analysis	All data analysis was done in R V4.3.2. The following open source programs were used: tidyverse, taxastand, devtools, phytools, ape, reshape2, ftolr, geiger, Taxonstand, ggsci, scales, taxize, ggtree, BAMMtools, hisse, diversitree, taxize, taxizedb, brew, and corHMM. Scripts used to perform analyses and plot results are available on GitHub 10.5072/zenodo.49427 (https://github.com/Suissajacob/FernNectaries). .

For manuscripts utilizing custom algorithms or software that are central to the research but not yet described in published literature, software must be made available to editors and reviewers. We strongly encourage code deposition in a community repository (e.g. GitHub). See the Nature Portfolio guidelines for submitting code & software for further information.

Data

Policy information about availability of data

All manuscripts must include a data availability statement. This statement should provide the following information, where applicable:

- Accession codes, unique identifiers, or web links for publicly available datasets
- A description of any restrictions on data availability
- For clinical datasets or third party data, please ensure that the statement adheres to our policy

The entire data generated in this study have been deposited in the GitHub database under accession code 10.5072/zenodo.49427 (https://github.com/Suissajacob/FernNectaries).

Research involving human participants, their data, or biological material

Policy information about studies with human participants or human data. See also policy information about sex, gender (identity/presentation), and sexual orientation and race, ethnicity and racism.

Reporting on sex and gender	NA
Reporting on race, ethnicity, or other socially relevant groupings	NA
Population characteristics	NA
Recruitment	NA
Ethics oversight	NA

Note that full information on the approval of the study protocol must also be provided in the manuscript.

Field-specific reporting

Please select the one below that is the best fit for your research. If you are not sure, read the appropriate sections before making your selection.

☐ Life sciences ☐ Behavioural & social sciences ☒ Ecological, evolutionary & environmental sciences

For a reference copy of the document with all sections, see nature.com/documents/nr-reporting-summary-flat.pdf

Ecological, evolutionary & environmental sciences study design

All studies must disclose on these points even when the disclosure is negative.

Study description	We used a multi-faceted approach to explore the evolution of nectaries in ferns, extra-floral nectaries in angiosperms, and plant associations in ants. The data collection process involved assembling four distinct datasets, each focusing on a specific aspect of the study, such as fern nectaries, angiosperm extra-floral nectaries, ant-plant interactions, and fern-feeding arthropod herbivores. These datasets were compiled from literature records, expert consultations, herbarium specimens, and previously published data. We then integrated robust phylogenies of ferns, flowering plants, and ants to use for ancestral character state estimation and downstream phylogenetic comparative methods.
Research sample	The data collection procedure for this study encompassed the compilation of four distinct datasets: extra-floral nectaries in angiosperms, nectaries in ferns, plant associations in ants, and fern-feeding arthropod herbivores. For the fern nectaries dataset, information was gathered through an extensive process involving literature records, expert consultations, and the observation of herbarium specimens.
Sampling strategy	Species were selected based on their presence in the largest phylogenies for each group (ferns, flowering plants, ants).
Data collection	Study species were selected based on their presence in the Fern Tree of Life project (FTOL) and relevant publications, with trait data verified using specimens from the Herbarium of the L. H. Bailey Hortorium (BH) at Cornell University. Additionally, growth habit information for ferns with nectaries was sourced from previously published data. The angiosperm extra-floral nectaries dataset utilized data from The World List of Plants with Extrafloral Nectaries, underwent manual correction of species names, and was subjected to phylogenetic reduction to facilitate downstream analysis. The ant-plant interaction dataset, sourced from Nelsen et al., 2018 underwent data filtering to focus on ants with specific plant associations, which were then coded based on their diet, foraging habits, and nesting locations. The fern-feeding arthropod herbivores dataset, drawn from Fuentes-Jacques et al., 2022, involved an indirect phylogeny-inference approach using TimeTree5 to build a chronogram of representative species within each genus of fern herbivores. Data were generated from JS Suissa with help from undergraduate research technicians as indicated in the Acknowledgments.

Timing and spatial scale	Data were generated 3 times per week each week from August 2022–March 2023. The data generation stage was continued until we exhausted information for all species in the respective phylogenies.
Data exclusions	No data were excluded from the analyses.
Reproducibility	All analyses can be reproduced using the available code and dataset.
Randomization	This was not necessary for the study. We aimed to compile species-specific data which should not necessitate randomization.
Blinding	This was not necessary for the study. We aimed to compile species-specific data which should not necessitate blinding.

Did the study involve field work? ☐ Yes ☒ No

Reporting for specific materials, systems and methods

We require information from authors about some types of materials, experimental systems and methods used in many studies. Here, indicate whether each material, system or method listed is relevant to your study. If you are not sure if a list item applies to your research, read the appropriate section before selecting a response.

Materials & experimental systems

n/a	Involved in the study	
☒	☐	Antibodies
☒	☐	Eukaryotic cell lines
☒	☐	Palaeontology and archaeology
☒	☐	Animals and other organisms
☒	☐	Clinical data
☒	☐	Dual use research of concern
☐	☒	Plants

Methods

n/a	Involved in the study	
☒	☐	ChIP-seq
☒	☐	Flow cytometry
☒	☐	MRI-based neuroimaging

Dual use research of concern

Policy information about dual use research of concern

Hazards

Could the accidental, deliberate or reckless misuse of agents or technologies generated in the work, or the application of information presented in the manuscript, pose a threat to:

No	Yes	
☒	☐	Public health
☒	☐	National security
☒	☐	Crops and/or livestock
☒	☐	Ecosystems
☒	☐	Any other significant area

Experiments of concern

Does the work involve any of these experiments of concern:

No	Yes	
☒	☐	Demonstrate how to render a vaccine ineffective
☒	☐	Confer resistance to therapeutically useful antibiotics or antiviral agents
☒	☐	Enhance the virulence of a pathogen or render a nonpathogen virulent
☒	☐	Increase transmissibility of a pathogen
☒	☐	Alter the host range of a pathogen
☒	☐	Enable evasion of diagnostic/detection modalities
☒	☐	Enable the weaponization of a biological agent or toxin
☒	☐	Any other potentially harmful combination of experiments and agents

Plants

Seed stocks	Relevant publications on the study species were identified through a comprehensive search of the scientific literature using keyword searches, citation tracking, and consultation of reference books and field guides. We corroborated trait data and generated additional observation data using herbarium specimens selected from the Herbarium of the L. H. Bailey Hortorium (BH) at Cornell University. We further gathered information on the growth habit of ferns bearing nectaries using previously published data from.
Novel plant genotypes	NA
Authentication	NA
